# Traceability of Pediatric Antibiotic Purchasing Pathways in Italy: A Nationwide Real-World Drug Utilization Analysis

**DOI:** 10.3389/fphar.2020.01232

**Published:** 2020-08-12

**Authors:** Janet Sultana, Gianluca Trifirò, Valentina Ientile, Andrea Fontana, Francesco Rossi, Annalisa Capuano, Carmen Ferrajolo

**Affiliations:** ^1^Department of Biomedical and Dental Sciences and Morphofunctional Imaging, University of Messina, Messina, Italy; ^2^Istituti di Ricovero e Cura a Carattere Scientifico Casa Sollievo della Sofferenza, San Giovanni Rotondo, Italy; ^3^Department of Clinical and Experimental Medicine, University of Campania “L. Vanvitelli”, Naples, Italy; ^4^Campania Regional Centre for Pharmacovigilance, Naples, Italy

**Keywords:** antibiotics, pediatric, self-medication, observational study, Italy

## Abstract

**Purpose:**

The aim of the present study was to describe the purchasing patterns of a set of antibiotics used exclusively in an out-patient pediatric setting in Italy using the Farma360 wholesale drug database (IQVIA Solutions Italy), identifying the proportion of medications which are not captured by Italian National Health Service (NHS) pharmacy claims databases and examining the implications of such findings from a public health and pharmaceutical policy perspective.

**Methods:**

Using a systematic approach, sixty-six antibiotic pediatric formulations were selected for the 5 most commonly used antibiotics in Italy in children and adolescents: amoxicillin in combination with clavulanic acid, amoxicillin, azithromycin, clarithromycin and cefixime. The Farma360 wholesale drug purchasing database was used to identify the yearly proportion of antibiotics not purchased based on NHS reimbursement in primary care from 2015–2017 at the national level. The relationship between product cost and purchase outside the NHS was assessed by a scatterplot. All analyses were stratified by geographic area: Northwest, Northeast, Central and Southern Italy.

**Results:**

The proportion of antibiotics not reimbursed by the NHS increased nationally from 24% in 2015 to 29% in 2017. The antibiotic with the highest proportion of purchases outside the NHS was amoxicillin, with almost two-thirds of all amoxicillin purchases in Southern Italy being made in this way in 2017. The relationship between antibiotic price and antibiotic purchase outside the NHS was almost linear for many geographic areas.

**Conclusions:**

This study showed that a large proportion of antibiotics with a pediatric formulation is purchased outside the NHS drug purchasing pathway, especially in Southern Italy, indicating that it is not possible to fully monitor drug utilization, including appropriateness, for these antibiotics. A better strategy is needed to improve drug utilization monitoring, such as better data collection or data linkage.

## Introduction

A meta-analysis of pediatric pharmacoutilization studies conducted in several countries around the world showed that around 60% of children are exposed to medications yearly, the majority of which (33%) were antibiotics (Clavenna and Bonati, 2009). This trend is also seen in Italy, with the prevalence of use in the community setting varying from 43% in Southern Italy to 70% in Northern Italy (Piovani et al., 2013). Although inappropriate antibiotic use is a widely acknowledged problem, the unnecessary use of antibiotics, especially in viral infections, still persists today. Indeed, one third of all antibiotics prescribed in the US are considered to be unnecessary, and it is estimated that half of all respiratory tract infections in the US are treated unnecessarily with an antibiotic (Fleming-Dutra et al., 2016). Inappropriate use of antibiotics in children is of high public health interest because of increasing antibiotic resistance, in addition to causing preventable adverse drug reactions and costs (Hersh and Kronman, 2017). The inappropriate use of antibiotics is driven by various factors which include but are not limited to poor prescribing practices and self-medication without a prescription (Lescure et al., 2018). Self-medication without a prescription is arguably an important point for public health intervention. All these issues highlight the need for systematic and thorough monitoring of antibiotic purchasing patterns. Indeed, previously published work shows the importance of studying antibiotic consumption through medication sales databases (Van Boeckel et al., 2014).

**Figure 1:**
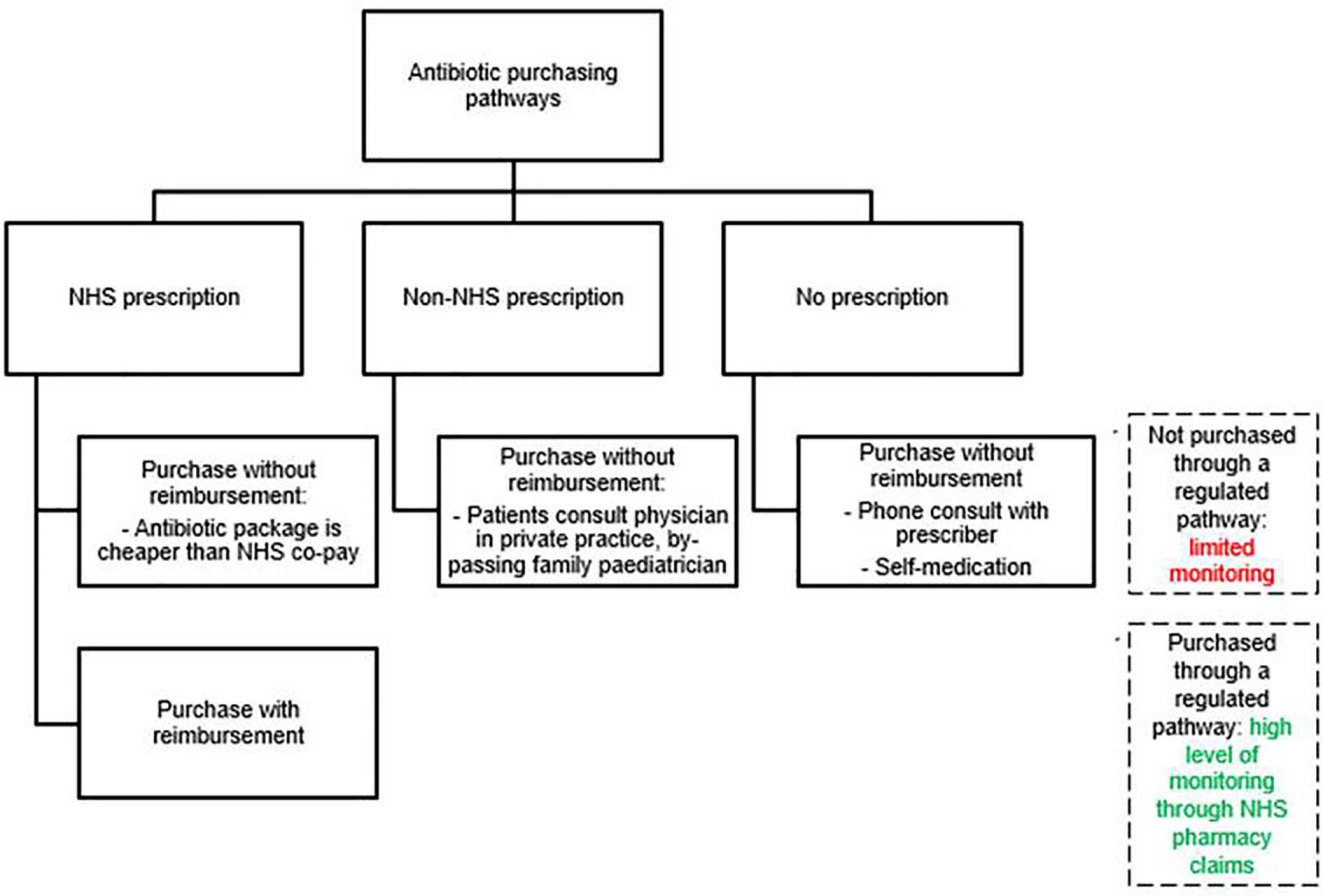
Antibiotic purchasing pathways in Italy. NHS, National Healthcare System.

Antibiotic purchasing in Italy can occur *via* several pathways, which can be summarized as the National Health Service (NHS) drug purchasing pathway and non-NHS drug purchasing pathway (**Figure 1**). A national report on antibiotic utilization in Italy showed that 17% of all antibiotic purchasing in the general population occurs outside the NHS drug purchasing pathways, i.e. is out-of-pocket (OsMed Report, 2019). This way of purchasing medications was twice as high in Northern Italy compared to Southern Italy (24% vs. 12%). However, there was no information on antibiotics used in children specifically. This constitutes an important knowledge gap concerning drug utilization trends in Italy among children, whether at the national level or by specific geographic area, as it is known that drug utilization in Italy has strong variations in different geographical area (Piovani et al., 2013).

Antibiotic purchasing pathways also have important implications for pharmacoepidemiology studies conducted using Italian NHS pharmacy claims databases, which leverage data concerning medications purchased through the NHS (Trifirò et al., 2019). Pharmacy claims data are very useful to study drug utilization compared to electronic medical records, because they are a step closer to confirming drug exposure, as patients having a prescription may not fill it at the pharmacy. However, pharmacy claims only capture medication dispensing based on the presence of an NHS prescription (Youngster et al., 2017). The inability to capture out-of-pocket purchasing of medications has often been cited as a limitation of such studies (Piovani et al., 2013) but the underestimation has never been quantified. The aim of the present study was to describe purchasing patterns of a set of antibiotics used exclusively in an outpatient pediatric setting in Italy, identifying the proportion of medications which are not captured by Italian NHS pharmacy claims databases and examining the implications of such findings from a public health and pharmaceutical policy perspective.

## Materials and Methods

### Setting

The present study is based in an Italian community setting, where pediatric primary care is provided by the family pediatrician (FP) within the NHS, for all children up to 14 years of age, thereafter by general practitioner (GP) (Ferrajolo et al., 2017). Therefore, the majority of prescriptions for children should be issued by NHS pediatricians in the community setting, using an NHS prescription. Medications prescribed through other medical specialists in their private practice would also be covered by the NHS, provided that they issue an NHS prescription which is used to purchase the medication. However, specialists in a private practice can also write non-NHS prescriptions, i.e. prescriptions which do not entitle patients to reimbursement.

In Italy, all antibiotics for systemic administration used in the primary care setting are covered by the NHS, either at directly the time of purchase or after the purchase by reimbursing the patient (Clavenna and Bonati, 2011). The patient is required to co-pay part of the medication price for all medications, unless they are exempt because of low income or because they have a diagnosis of a chronic disease. In addition to the co-pay, patients are required to pay the difference between a generic and non-generic medication if they choose to buy a non-generic medication. Practically, all antibiotics for systemic use in Italy should be captured by NHS claims data if they are purchased with an NHS prescription and then reimbursed by the NHS after a claim is made, in line with national antibiotic dispensing policies. Purchases made with an NHS prescription and captured as NHS pharmacy claims were therefore considered to denote antibiotics purchased within a nationally approved and regulated prescribing pathway with a high degree of transparency and traceability. All other types of antibiotic purchases were considered to be made in an unidentifiable prescribing pathway and cannot be monitored. Such purchasing pathways have very limited transparency and traceability.

### Data Sources

Nationally representative pharmacy sales data from Farma360, a database owned by a commercial company, IQVIA, was extracted from 2015 to 2017 using IMS Dataview 7.0. This database contains aggregate information on the number of drug packages sold to and bought from a sample of community pharmacies from four macro-areas in Italy covering all Italian regions. This sample comprises a minimum of 5 and a maximum of 10 community pharmacies in every region. The data collected allows the distinction between medications which are not sold through the NHS drug purchasing pathway (i.e. may or may not be purchased with a prescription, NHS or otherwise, but not reimbursed by the NHS) from those which are (i.e. definitely purchased with an NHS prescription with subsequent reimbursement). Drug data in IMS is coded using the Anatomic Therapeutic Chemical classification code (ATC) and by a National Drug Code (NDC), known in Italy as *Autorizzazione all’Immissione in Commercio* code. The NDC is specific for strength, formulation, route of administration and manufacturer. The dispensing information in the database contains the geographical area where the drug was purchased. This database does not contain patient-level or aggregate-level information on patient age, sex and medical history. To our knowledge, this is the first paper to publish findings from the use of the Farma360 database in Italy.

### Study Population

The demographic and socioeconomic status of the catchment areas was extracted from publically available information on the Italian National Statistics Office using the latest available data (https://www.istat.it/en/). Specifically, the age and sex distribution for children aged up to 14 was extracted across all catchment areas up until 2019. To describe the socioeconomic status of the parents/families within the catchment areas, the mean income per family in each Italian macro-area in 2017, extracted from the latest available data (2017) of the Italian National Statistics Office. In addition, information on the number of persons aged 15 and over with specific categories of education level was extracted from the Italian National Statistics Office for the year 2019. The education levels available were: completion of primary school, secondary school, vocational school, high school or tertiary education (graduate or post-graduate degree).

### Study Drugs

Medicinal products intended exclusively for pediatric use available on the market between 2015 and 2017 were identified systematically by a clinical pharmacologist specialising in pediatric pharmacology. First, the five most commonly used antibiotics in the Italian pediatric population were identified from the Italian National Report on Drug Utilization report (OsMed, 2015): amoxicillin, amoxicillin–clavulanic acid combination (i.e. co-amoxiclav) cefixime, azithromycin and clarithromycin. Secondly, the NDCs of all the medicinal products marketed in Italy corresponding to these five antibiotics were identified using a national medicines database, Farmadati. This database contains information (including NDC) concerning the medicinal products currently available on the market in Italy as well as for those which have been discontinued. A total of 288 NDCs referring to selected antibiotics were identified. Thirdly, among the 288 NDCs, formulations intended specifically for children were identified based on their formulations (e.g. syrups, solutions/power for reconstitution, chewable tablets, suppositories etc.) and strength (i.e., the amount of active substance per package), in particular those having lower strength intended for children and the indication “for children” as specified on the packaging and leaflet. After this phase of screening, 66 NDCs specific for medicinal products with pediatric formulations were identified. The list of medications included in the study is available in **Supplementary Material Table 1**. The prices for these medicinal products fixed by the NHS were obtained separately for each year from the Italian National Drug Agency website, to account for potential change in price (https://www.aifa.gov.it/liste-di-trasparenza).

### Statistical Analysis

All statistical analyses were conducted exclusively considering medicinal products with pediatric formulations. The percentage of medicinal product units purchased through the NHS drug purchasing pathway and the percentage of medicinal product units which were not purchased in this way was estimated for the 66 medicinal products having a pediatric formulation using 2015, 2016, and 2017 data at the national level. Results were grouped by fifth level ATC and were also graphically represented by horizontal stacked bar charts.

The distribution of purchases made through the NHS drug purchasing pathways and other purchasing pathways in Italy was then stratified by four macro-areas, i.e. Northeast Italy, Northwest Italy, Central Italy and Southern Italy. The regions included in each macro-area are reported in **Supplementary Material Table 2**. This was done as drug utilization patterns in Italy are known to vary significantly in these macro-areas (OsMed Report, 2015); the population living in the same macro-area shares the same demographic characteristics influencing disease pattern and drug intake.

The proportion of antibiotics purchased outside the NHS drug purchasing pathways (i.e. number of antibiotic purchases without NHS reimbursement divided by all antibiotic purchases) were described using box plots. The distribution of single drug product purchasing patterns was evaluated, based on the NDC and clustered at the ATC fifth level, and reported in box plots as minimum, first quartile, median, third quartile, and maximum number of purchases for each fifth level ATC. The central rectangle spans the first quartile to the third quartile (i.e. the interquartile range or IQR). The segment inside the rectangle shows the median and “whiskers” (above and below each box) show the locations of the minimum and maximum. These descriptive analyses were carried out at the national level and by geographic area. The presence of a statistical difference between yearly median percentage of antibiotic purchase without prescription, as well as between the total number of antibiotics bought with and without an NHS prescription was tested using the Friedman rank sum test, i.e. non-parametric repeated measures ANOVA.

To assess the relationship between antibiotic purchases occurring outside the NHS drug purchasing pathways and medicinal product prices, a scatterplot fitted through a Locally Estimated Scatterplot Smoothing (LOESS) curve was carried out overall and stratified by geographic area (Cleveland et al., 1979). Furthermore, to statistically quantify the strength (and the direction) of the relationship between the purchase of any of the five study antibiotics outside the NHS antibiotic purchasing pathway and costs, Spearman’s rank correlation coefficients were estimated.

All statistical analyses and plots were carried out using R Foundation for Statistical Computing (version 3.6, packages: ggplot2, gridExtra). P-values lower than 0.05 were considered to denote statistical significance.

## Results

The demographic and socioeconomic status of the population in the catchment areas are presented in **Supplementary Material Table 3** and **Supplementary Material Figures 1** and **2**. These data show that there is no difference in the sex and age distribution from Northeast/Northwest to Southern Italy, while there is a strong gradient of decreasing mean family income from Northeast/Northwest to Southern Italy as well as decreasing education levels from Northeast/Northwest to Southern Italy for persons aged 15 and over.

**Figure 2:**
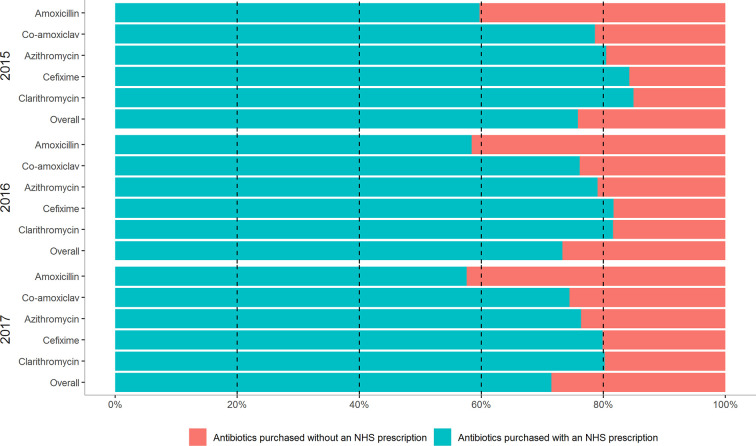
Antibiotic purchasing trends at the national level in Italy for pediatric formulations of the top five antibiotics used in children by calendar year.

Overall, 66 medicinal products corresponding to the 5 most commonly used antibiotics were selected. On average, 27% of any of the 5 antibiotics medication were purchased outside the NHS drug purchasing system from 2015 to 2017. There was a modest increase in the purchase of antibiotics outside the NHS system, from 24% in 2015 to 29% in 2017. There were notable differences in the trends for single active ingredients. The antibiotic drug most commonly purchased out-of-pocket was amoxicillin (approximately 40% over the 3 study years), followed by co-amoxiclav, azithromycin, cefixime and clarithromycin (approximately 20% from 2015-2017). These trends remained constant over the study period (**Figure 2**).

**Figure 3:**
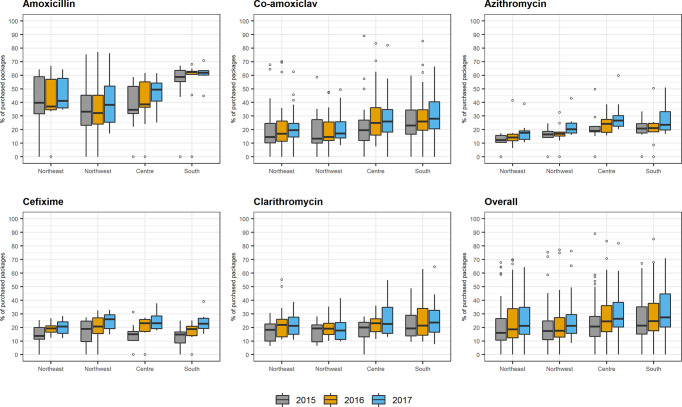
Box plots of percentage of packages distribution purchased without a prescription for the top five antibiotics used in children by geographic area and by calendar year.

On stratifying results by geographic area, a temporal and geographic trend in the purchase of antibiotics for children outside the NHS drug purchasing pathway was often seen based on the median and IQRs (**Figure 3**), with respective p-values reported in **Supplementary Material Table 4**. Amoxicillin was the drug most commonly purchased outside the NHS purchasing pathway throughout the study period. This was more marked in Southern Italy, where almost two thirds of this drug were purchased outside the NHS purchasing pathway from 2015 (58.7%; IQR: 55.2–63.6%) to 2017 (61.9%; IQR: 60.1–63.3%). The greatest increase in out of pocket purchase for amoxicillin over time was seen in Central Italy, where this increased from 34.6% (IQR: 31.8–51.9%) in 2015 to 49.3% (IQR: 41.0–54.2%) in 2017 (p-value: 0.011). Medications containing azithromycin were among the least commonly purchased drugs outside the NHS, ranging from 17.65% (IQR: 12.9–19.0%) to 23.5 (IQR: 20.8–33.2%) from Northeast and South Italy in 2017, respectively. The overall trend for all five antibiotics suggested that there was a slightly greater tendency to purchase these drugs outside the NHS purchasing pathways in the South of Italy compared to the North. For example, in 2017, 21.2% (IQR: 14.9–34.8%) and 21.2% (IQR: 15.7–29.4%) of antibiotics were purchased outside the NHS purchasing pathway in North eastern and North western Italy, respectively, while 26.4% (IQR: 20.3–38.4%) and 27.3% (IQR: 20.4–44.6%) did this in central and Southern Italy, respectively. The distributions of the number of packages underlying these proportions for antibiotics purchased outside the NHS purchasing pathways and those purchased within the NHS drug purchasing pathway are consistent (data not shown).

**Figure 4:**
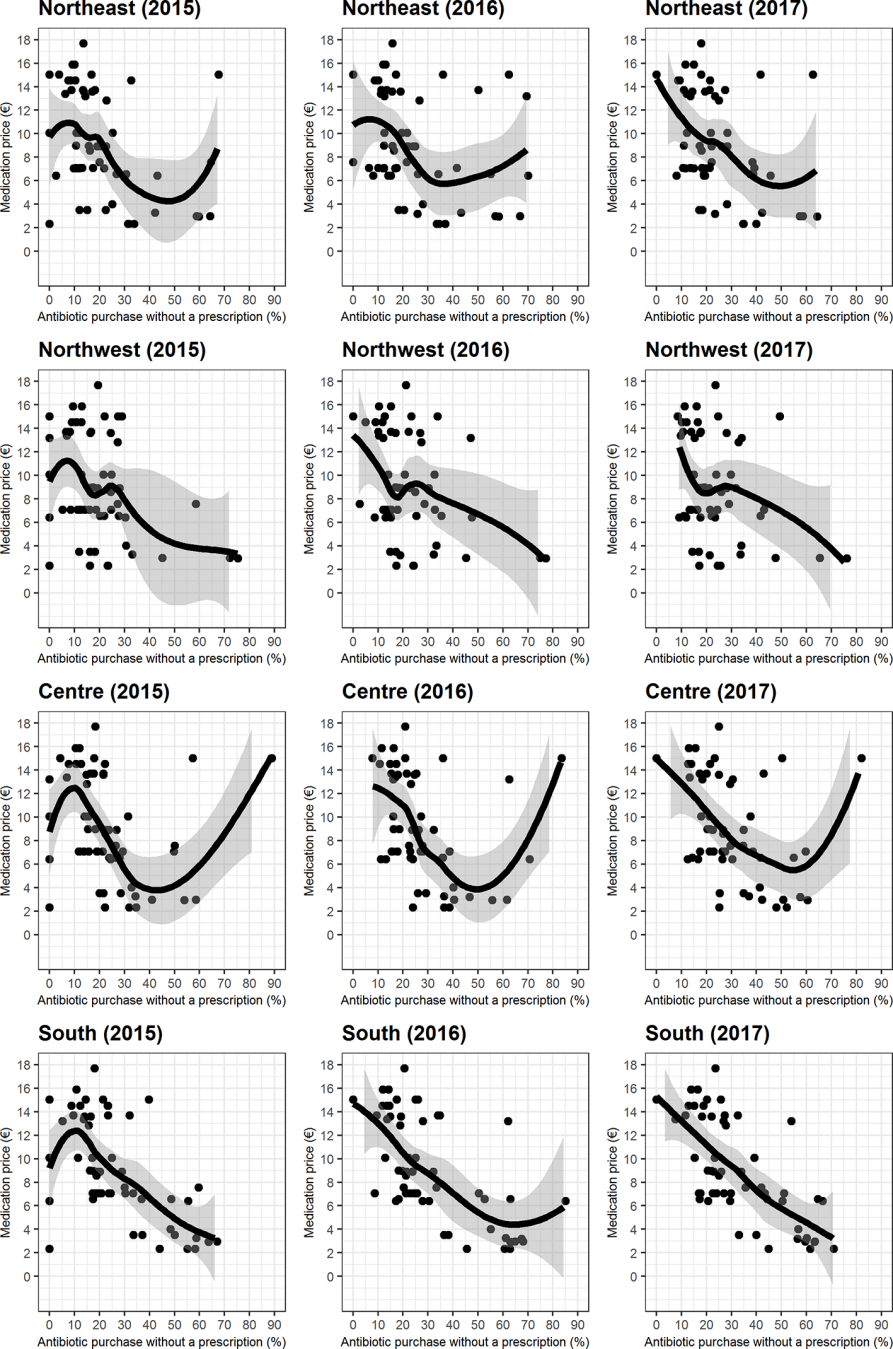
Relationship between medication price and the percentage of the top five pediatric antibiotic purchases without a prescription, along with Locally Estimated Scatterplot Smoothing curves.

As shown by the scatterplots and LOESS curves (**Figure 4**), the relationship between antibiotic price and antibiotic purchase outside the NHS drug purchasing pathway was markedly not linear (u-shaped) in Central Italy and was almost linear for the other geographic areas (**Figure 4**). All the relationships seen between the purchase of antibiotics outside the NHS drug purchasing pathway suggested that the lower the prices, the higher the proportion of purchase outside NHS pathway. This is shown by Spearman’s coefficient, which was always negative (**Supplementary Material Table 5**). The strongest trends were seen in Southern Italy, where a statistically significant negative correlation was seen in each year (<0.0001). Furthermore, the strength of this correlation increased over the years in Southern Italy, from a Spearman’s coefficient of −0.58 in 2015 to −0.72 in 2017. The weakest correlations were seen in Northwest Italy, where the Spearman’s coefficient was −0.23 in 2017, suggesting that the relationship between drug price and the purchase of antibiotics outside the NHS drug purchasing pathway was less strong in this area.

## Discussion

The present study is the first to describe the extent of pediatric antibiotic purchasing patterns in Italy as occurring through a regulated and, therefore, traceable and transparent pathway or otherwise. We have estimated that at the national level, around one third of antibiotics for children were purchased outside the NHS drug purchasing pathway and this proportion increased modestly from 2015 to 2017. This finding is of relevance to all pharmacoepidemiologic studies conducted using Italian claims databases (De Bie et al., 2015; Piovani et al., 2016; Putignano et al., 2017; Ferrajolo et al., 2019), because our findings suggest that these studies may underestimate the use of antibiotics at least by 30%; for specific antibiotics in certain catchment areas, for example, amoxicillin in Southern Italy, this may be as high as 60%. While the extent of underestimation may vary for specific drug classes and for different populations, this is the first time that the issue is quantified; previous studies only allude to it, even suggesting that it is likely to be minimal (Piovani et al., 2013). The main strength of these Italian claims-based studies is their population-based setting, where all contact with the NHS is captured as pharmacy claims or hospital claims, because Italy has a universal healthcare system (Trifirò et al., 2019). Indeed, several studies have used these claims databases to evaluate antibiotic use and safety in children (Lapi et al., 2010; Piovani et al., 2013; Ferrajolo et al., 2019).

The antibiotics included in this study were the most commonly used in pediatric patients in the year before the study commenced (OsMed Report, 2015) and remain the most commonly used antibiotics in 2017 (OsMed Report, 2018). The purchase of antibiotics outside the NHS drug purchasing pathway was always higher in Southern Italy, accounting for approximately two-thirds of the purchase of amoxicillin, with the implication being that it is not known whether this volume of antibiotic purchasing occurred with or without any kind of prescription. Indeed, there is a higher use of antibiotics among children in the South of Italy compared to the North (OsMed Report, 2018). This can be due to a range of reasons, the impact of which unfortunately, cannot be ascertained. The purchase of antibiotics outside the NHS drug purchasing pathway may indicate the use of a non-NHS prescription from a pediatrician in a private practice, or even of an NHS prescription without request for reimbursement. It may also indicate antibiotic purchase without any prescription at all. Non-NHS prescriptions, often written by a specialist, would not make patients eligible for reimbursement. On the other hand, the use of NHS prescriptions which are not followed by a claim for reimbursement may occur because, despite being eligible for full or partial reimbursement, parents choose not to file a claim as the medication price is very cheap, especially if the co-pay costs as much as the medication reimbursement. This could be the case for the amoxicillin, which is the cheapest antibiotic among our study drugs. Indeed, the proportion of amoxicillin purchased outside NHS pathway is much higher than that observed for azithromycin, clarithromycin and cefixime. In addition, amoxicillin is the first-line antibiotic therapy for common infections in childhood, such as otitis, while macrolides are recommended only in case of allergy to beta-lactam antibiotics (Marchisio et al., 2019). Parents may therefore be more familiar in using this antibiotic without a prescription as compared to other less commonly used antibiotics because it is likely to have been previously prescribed. Indeed, a study conducted using the Lombardy claims database showed that amoxicillin is by far the most commonly used antibiotic among children compared to co-amoxicillin, azithromycin, clarithromycin and cefuroxime (Clavenna et al., 2010). From a pharmacological perspective, all the antibiotics included in the study are considered broad-spectrum antibiotics in clinical practice (Gerber et al., 2017). This is therefore unlikely to have affected the degree of out-of-pocket purchases for the study drugs. The out-of-pocket purchase of antibiotics may also indicate the purchase of antibiotics after phone consultation with prescribers. Indeed, a qualitative study in Italy showed that more than half of parents admitted to giving antibiotics to their children only after a phone consult with the prescriber, i.e. without an examination and without a prescription, although this is not in line with Italian prescribing policies (Bert et al., 2017). It should be noted that in Italy, antibiotics should only be purchased with a prescription, whether from an NHS prescriber or a prescriber from a private practice. Nevertheless, the OsMed national report on antibiotic use in 2017 showed that 17% of all antibiotics in Italy are purchased outside the NHS drug purchasing pathway. However, this analysis was not specifically conducted on such purchasing trends in children (OsMed Report, 2018).

The reason behind the purchase of antibiotics outside the NHS is difficult to ascertain using any source of secondary data. Explanations of antibiotic purchasing behaviour outside of NHS purchasing system can only be obtained *via* survey responses of the parents making such purchases. In a survey of over 400 parents of primary school children in Central Italy, one-third of all parents admitted to using antibiotics without a prescription, while almost 25% of parents who had never purchased antibiotics without a prescription said they would be willing to do so (Napolitano et al., 2013). These figures are in line with national findings from the present study that 24–29% of selected antibiotic purchasing occurred without an NHS prescription from 2015 to 2017, further increasing the plausibility that the purchases without an NHS prescription reflect self-medication. A more recent survey of parents’ knowledge and attitudes concerning antibiotics was published in a wider catchment area covering cities from Northern, Central and Southern Italy (Bert et al., 2017). A quarter of parents in these areas admitted to buying antibiotics for their children without a prescription although no information on antibiotic preference was provided (Bert et al., 2017). Indeed, self-medication in Italy has been shown to be as high as 50% among a sample of almost 700 parents in central Italy (Garofalo et al., 2015). The importance of monitoring antibiotic use diligently and systematically is primarily related to the risk of antibiotic resistance. In addition, misuse and over-use of antibiotics is associated with potentially serious acute as well as long-term risks such as those attributed to imbalances in gut microbiota, called dysbiosis. In particular, frequent and inappropriate antibiotic use during infancy may determine short- and long-term effects on the diversity and composition of the gut microbiota which has been associated with the onset of obesity, diabetes, asthma, allergy and autoimmune disease later life (Vangay et al., 2015).

Another important finding of the present study was that antibiotic purchase outside the NHS drug purchasing pathway was generally correlated to product price, with lower prices correlating to higher non-prescription purchases. However, we also found that this correlation differs markedly from north to south Italy. The present study shows that the implementation of any policies aiming to regulate antibiotic purchases would have to take geographic area into account: it is only in Southern Italy where there was a strong correlation between increasing drug prices and decreasing purchases outside the NHS drug purchasing pathway. This is likely due to the known socioeconomic gradient in Italy, as Northern regions have a better socioeconomic status than Southern ones. As a result, persons in the South are likely to be less willing to pay a higher price for antibiotics when purchasing them out-of-pocket, i.e. without NHS reimbursement. Indeed, one of the World Health Organisation’s policy recommendations to reduce antibiotic resistance is to find a method of payment and reimbursement that promotes compliance to treatment guidelines and discourages irrational use (Leung et al., 2011). It may be worth adding that the promotion of systematic data collection on all antibiotic purchases is important in order to monitor whether and to what extent such purchases occur within regulated and appropriate purchasing pathways. In 2017, the Italian Ministry of Health launched a national plan to reduce antibiotic resistance, with one of the aims being the reduction of systemic antibiotic use by at least 10% in the community setting (Italian Ministry of Health, 2017). While the commitment to improve antibiotic use surveillance in Italy has improved, as can be seen by the yearly publication of the OsMed national report on antibiotic use in Italy and in each region, it is not year clear whether the objective of reducing unnecessary antibiotic use has been achieved. If national plans to reduce antibiotic resistance are to be effective, it is essential that the various stakeholders, such as family pediatricians, specialists, pharmacists, and parents, are continuously educated on the topic and work together by respecting healthcare policies, in their respective sphere.

The present study has several strengths. It is the first to compare antibiotic purchasing pathways in Italian pediatric patients longitudinally, i.e. non-NHS drug purchasing pathway and the NHS drug purchasing pathway. This was done both at the national level, as well as in specific geographic areas. The data source used to do this, the Farma360 wholesale drug purchase database, is one of the few that contains information on non-NHS prescription purchases. A similar database has been used to describe global antibiotic consumption, showing that medication purchasing databases are both useful and reliable (Van Boeckel et al., 2014). The analysis on the correlation between drug cost and purchases outside the NHS drug purchasing pathway was important to demonstrate that low cost is not always the main driver of antibiotic purchasing outside a purchasing pathway which offers drug reimbursement. We were also able to show that the relationship between drug cost and antibiotic purchases outside the NHS drug purchasing pathway varies across geographic regions. It is possible, and indeed probable, that such a correlation between out-of-pocket medication purchases and price also exists for other medications. Similar studies at the national level should be conducted to assess this, as it could have important implications from a public health and pharmaceutical policy perspective.

This study also has some limitations. We have little information concerning the purchase of antibiotics outside the NHS drug purchasing pathway: we cannot know whether the purchase was made with or without a prescription issued by physicians in their private practice. We did not assume that such purchases definitely constituted non-prescription purchases and could not investigate the matter further. We also did not have information on the indication of use for the selected antibiotics. However, evaluating antibiotic appropriateness based on indication was beyond the scope of this study, which was limited to describing the purchases of antibiotics for pediatric use in terms of transparency and traceability at a high level. Finally, we did not have any patient-level information and were therefore not able to describe the population purchasing the study drugs.

## Conclusion

Almost a third of the top five most commonly used antibiotics with pediatric formulations are purchased out-of-pocket. For amoxicillin, this is as high as 60% in the South of Italy. As a result, pharmacoepidemiologic studies using Italian claims databases, which leverage the Italian NHS reimbursement system, risk underestimating antibiotic use among children significantly. This may lead to exposure misclassification and potential bias. The private purchase of these drugs increases with decreasing medication price, especially in Southern Italy although the correlation was not strongly linear. Lastly, our results could suggest the need for better monitoring of antibiotic use within the NHS in order to reduce inappropriate antibiotic use which would in turn reduce antibiotic-resistance. A strategy is needed to improve antibiotic utilization monitoring, with special emphasis on those drugs purchased outside the formal NHS pathway, which are more likely to be inappropriately prescribed. Public health interventions aimed at preventing antibiotic purchasing without a prescription among healthcare professionals and patients alike, are urgently needed.

## Data Availability Statement

The datasets presented in this article are not readily available because data is not available to third parties as it was acquired from data providers with restrictions on data sharing. Requests to access the datasets should be directed to Carmen.ferrajolo@unicampania.it.

## Ethics Statement

Ethical review and approval was not required for this study accordance with the local legislation and institutional requirements. Written informed consent for participation was not required for this study in accordance with the national legislation and the institutional requirements.

## Author Contributions

CF and GT conceived the study. VI and AF carried out the data analysis. JS, GT, VI, AF, AC, and CF interpreted the data. JS and CF prepared the first draft of the paper. All authors contributed to the article and approved the submitted version.

## Funding

This study was funded by the Italian Ministry for Research and Education for the project “Music USe In Children - MUSIC”, awarded to CF as part of the SIR funding program (RBSI14X2S5).

## Conflict of Interest

GT has received funding for studies not related to the present paper: he is principal investigator of observational studies funded by various pharmaceutical companies (e.g. Amgen, AstraZeneca, Daiichi Sankyo, IBSA) to University of Messina; he is also the scientific coordinator of the Master’s program “Pharmacovigilance, pharmacoepidemiology and pharmacoeconomics: real world data evaluations” at University of Messina which receives unconditional funding by several pharmaceutical companies.

The remaining authors declare that the research was conducted in the absence of any commercial or financial relationships that could be construed as a potential conflict of interest.
