# Supplementary material for: Traceability of Pediatric Antibiotic Purchasing Pathways in Italy: A Nationwide Real-World Drug Utilization Analysis
Source: Front Pharmacol. 2020 Aug 12;11:1232. doi: 10.3389/fphar.2020.01232 (PMC7435014; doi:10.3389/fphar.2020.01232)
Supplement: Supplementary file 1 [file DataSheet_1.pdf]

Supplementary Material

Table 1: Medications with pediatric formulations included in the study.

Active ingredient	NDC
Amoxicilin	023086061; 023086097; 23086162; 023097013; 23097037; 023760034; 023966106; 033151034; 034812014
Co-amoxiclav	026089110; 026089122; 26089134; 026089146; 026138204; 026138216; 026138228; 026138230; 026141200; 026141212; 026141224; 036980148; 036980175; 036980201; 037350028; 042221022; 042221046; 042856031; 037526047; 037747019; 037747021; 037954043; 039785035; 040488025
Cefixime	027127101; 027134105; 027152091; 028855056; 038905081; 039791013; 039976028; 041391083
Clarithromycin	027370067; 027370117; 027529041; 027529104; 027530068; 027530120; 037407044; 037446010; 037446022; 037456011; 037456023; 037527049; 037527090; 041557051; 041557099
Azithromycin	027860028; 027897026; 027948052; 028177020; 037972054; 037973056; 038878056; 038929042; 039852052

Abbreviation: NDC – national drug code

Table 2: Categorisation of regions into macro-areas.

Macro-area	Region
Central Italy	Lazio
	Marche
	Toscana
	Umbria
Northeast Italy	Emilia Romagna
	Friuli Venezia Giulia
	Trentino Alto Adige
	Veneto
Northwest Italy	Liguria
	Lombardia
	Piemonte
	Valle d'Aosta
Southern Italy	Abruzzo
	Basilicata
	Calabria
	Campania
	Molise
	Puglia
	Sardegna
	Sicilia

Table 3: Demographics of the Italian pediatric population in North-west Italy as of 1st January 2019, extracted from the Italian National Statistics Office website.

	**Northwest Italy**			**Northeast Italy**			**Central Italy**			**Southern Italy**		
**Age**	**M** (N=1,082,034)	**F** (N=1,021,027)	**M + F (%)** (N=2,103,061)	**M** (N=794,498)	**F** (N=750,082)	**M + F (%)** (N=1,544,580)	**M** (N=797,712)	**F** (N=752,967)	**M + F (%)** (N=1,550,679)	**M** (N=1,421,543)	**F** (N=1,342,352)	**M + F (%)** (N=2,763,895)
0	58,547	55,711	114,258 (5.4)	44,042	41,362	85,404 (5.5)	42,605	40,335	82,940 (5.3)	80,234	75,451	155,685 (5.6)
1	62,255	59,076	121,331 (5.8)	45,752	43,009	88,761 (5.8)	44,661	42,736	87,397 (5.6)	83,418	79,015	162,433 (5.9)
2	64,415	61,397	125,812 (6.0)	47,474	45,014	92,488 (6.0)	47,691	45,028	92,719 (6.0)	84,728	80,273	165,001 (6.0)
3	67,123	62,814	129,937 (6.2)	48,658	46,345	95,003 (6.1)	48,752	46,398	95,150 (6.1)	86,854	82,093	168,947 (6.1)
4	68,822	65,483	134,305 (6.4)	50,302	47,798	98,100 (6.3)	51,108	48,136	99,244 (6.4)	89,159	83,612	172,771 (6.2)
5	70,464	66,404	136,868 (6.5)	51,473	48,923	100,396 (6.5)	52,249	49,018	101,267 (6.5)	89,352	85,114	174,466 (6.3)
6	73,209	69,024	142,233 (6.8)	53,797	50,632	104,429 (6.8)	54,429	52,085	106,514 (6.9)	93,792	88,291	182,083 (6.6)
7	74,734	69,925	144,659 (6.9)	54,551	51,671	106,222 (6.9)	55,303	52,694	107,997 (7.0)	95,873	90,476	186,349 (6.7)
8	76,688	71,914	148,602 (7.1)	56,522	53,038	109,560 (7.1)	56,786	53,606	110,392 (7.1)	98,431	93,121	191,552 (6.9)
9	78,229	74,164	152,393 (7.2)	57,021	53,871	110,892 (7.2)	57,402	53,695	111,09 7 (7.2)	100,189	94,636	194,825 (7.0)
10	78,580	74,243	152,823 (7.3)	57,572	54,320	111,892 (7.2)	58,065	54,929	112,99 4 (7.3)	101,495	96,500	197,995 (7.2)
11	78,045	73,305	151,350 (7.2)	57,154	53,982	111,136 (7.2)	58,338	54,309	112,647 (7.3)	101,935	96,802	198,737 (7.2)
12	77,932	73,324	151,256 (7.2)	57,007	53,760	110,767 (7.2)	57,361	54,091	111,45 2 (7.2)	104,019	97,177	201,196 (7.3)
13	76,643	71,811	148,454 (7.1)	56,641	52,923	109,564 (7.1)	56,428	52,982	109,410 (7.1)	104,804	98,467	203,271 (7.3)
14	76,348	72,432	148,780 (7.1)	56,532	53,434	109,966 (7.1)	56,534	52,925	109,459 (7.1)	107,260	101,324	208,584 (7.5)

Abbreviations: F – females; M – males

Figure 1: Mean income per family in each Italian macro-area in 2017, extracted from the latest available data of the Italian National Statistics Office.

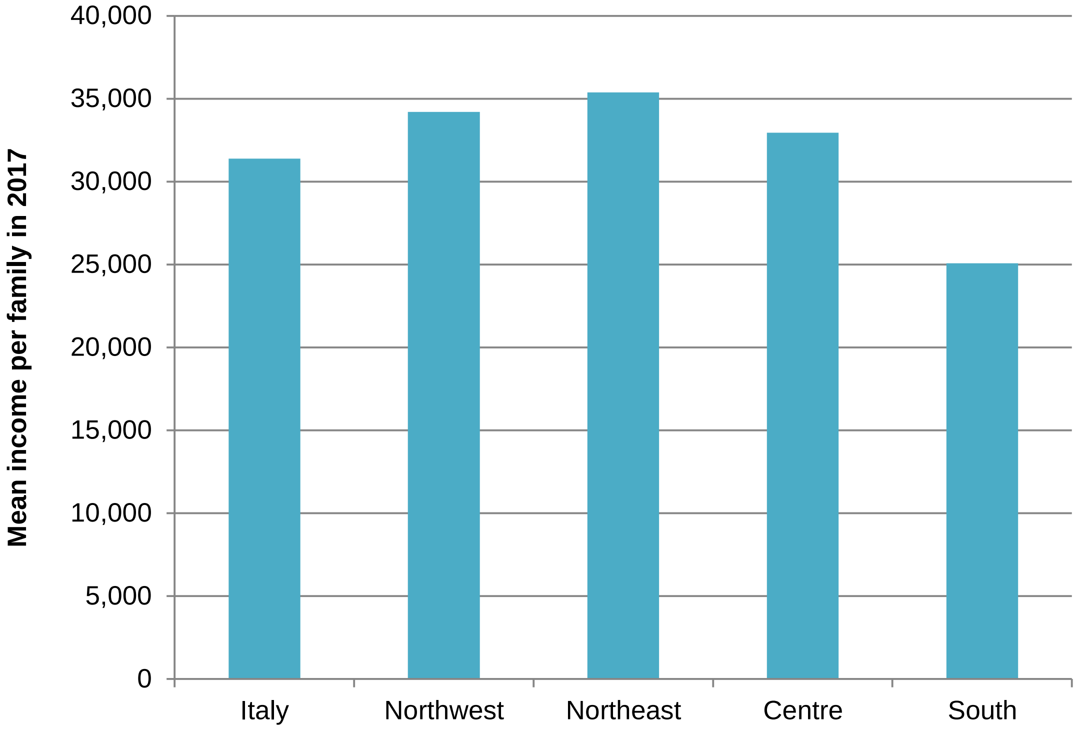

Figure 2: Number of persons aged 15 and over with specific categories of education level, extracted from the Italian National Statistics Office for the year 2019.

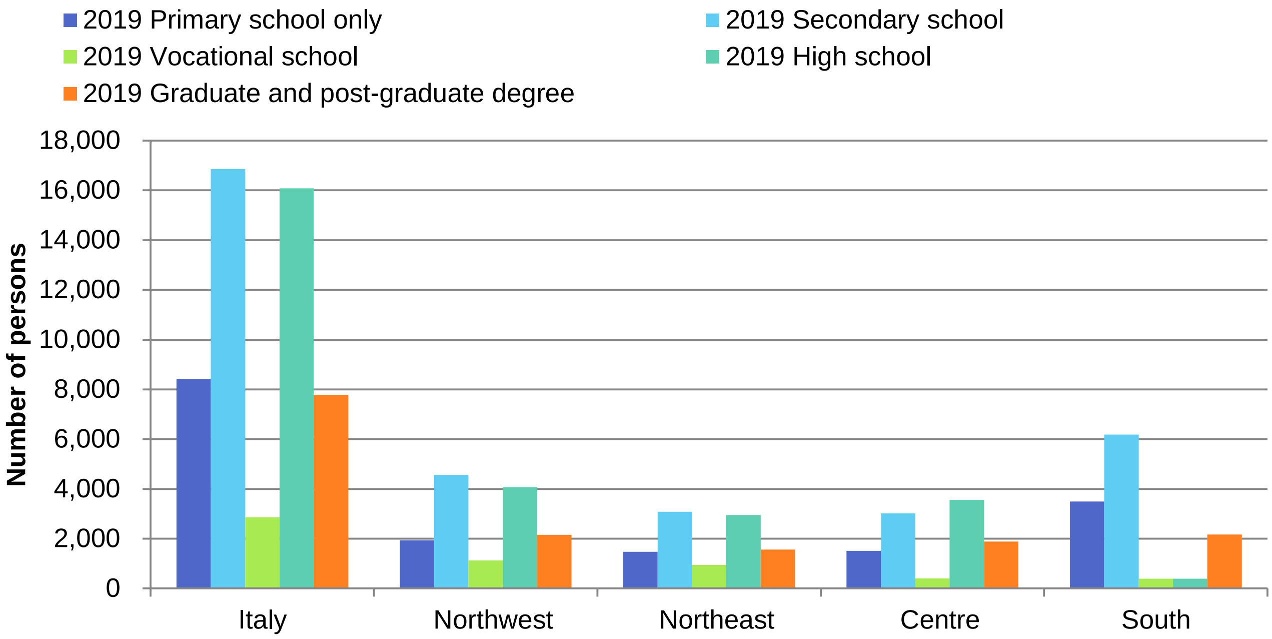

Table 4: Median proportion of antibiotic purchases without an NHS prescription, stratified by geographic area.

		Median (IQR)			
**Antibiotic**	**Area**	**2015**	**2016**	**2017**	**p-value***
Amoxicillin	Northeast	39.80 (31.70-58.90)	37.00 (34.50-57.00)	41.10 (36.00-57.62)	0.6065
	Northwest	33.10 (23.20-45.20)	32.20 (24.10-45.30)	38.20 (25.33-52.05)	0.0930
	Centre	34.60 (31.80-51.90)	38.60 (36.40-55.10)	49.35 (40.97-54.25)	0.0111
	South	58.70 (55.20-63.60)	62.40 (60.60-62.90)	61.85 (60.05-63.32)	0.1353
Co-amoxiclav	Northeast	14.60 (10.12-24.62)	17.00 (11.50-26.35)	19.60 (14.55-24.60)	0.0884
	Northwest	13.35 (10.07-27.33)	14.45 (11.93-25.50)	17.20 (13.75-25.77)	0.0010
	Centre	19.55 (11.85-27.00)	24.90 (15.95-36.10)	25.85 (18.15-34.75)	0.0176
	South	22.95 (16.52-34.40)	25.90 (19.50-34.47)	27.95 (20.57-40.50)	0.0080
Azithromycin	Northeast	12.30 (10.50-15.70)	14.10 (11.83-16.67)	17.65 (12.85-18.95)	0.0439
	Northwest	16.50 (14.10-18.60)	17.00 (15.20-18.20)	20.30 (17.45-24.77)	0.0076
	Centre	19.10 (18.30-22.30)	24.25 (18.03-27.40)	26.55 (22.43-30.27)	0.0098
	South	20.70 (17.50-24.40)	21.10 (18.30-24.50)	23.50 (19.75-33.20)	0.0724
Cefixime	Northeast	13.70 (11.35-19.90)	19.20 (16.45-21.12)	20.55 (15.58-24.10)	0.0421
	Northwest	18.90 (9.52-24.55)	20.70 (15.50-27.10)	26.00 (19.02-29.30)	0.0155
	Centre	14.85 (10.43-17.02)	23.10 (17.05-25.85)	23.05 (19.03-28.35)	0.0155
	South	14.80 (8.55-16.65)	18.80 (13.85-20.75)	22.70 (19.17-26.60)	0.0025
Clarithromycin	Northeast	18.20 (10.00-22.60)	21.90 (13.00-25.90)	21.20 (14.90-27.50)	0.0023
	Northwest	19.20 (9.40-21.80)	19.10 (14.80-23.00)	17.70 (11.20-23.60)	0.0073
	Centre	19.90 (13.07-23.78)	23.00 (16.90-26.30)	22.50 (15.60-34.80)	0.0079
	South	19.20 (13.70-28.90)	21.30 (14.30-33.90)	23.60 (16.50-32.50)	0.0136
Overall	Northeast	15.95 (10.55-26.43)	18.60 (12.40-33.67)	21.20 (14.90-34.75)	0.0469
	Northwest	17.40 (11.00-24.80)	17.50 (12.77-27.25)	21.20 (15.65-29.35)	0.0011
	Centre	20.60 (13.35-28.12)	24.50 (16.90-36.00)	26.40 (20.25-38.40)	0.0004
	South	21.30 (15.10-35.15)	24.60 (17.60-37.70)	27.30 (20.35-44.55)	0.0039

*p-values from Friedman rank sum test (i.e. non-parametric repeated measures ANOVA). **Abbreviation**: IQR: Interquartile range (i.e. first-third quartiles)

Table 5: Correlation between percentage of antibiotic purchases for amoxicillin, co-amoxiclav, cefixime, clarithromycin and azithromycin outside the NHS drug purchasing pathway and drug price, stratified by geographical area.

**Geographical area**	**Year**	**Spearman correlation**	**p-value**
Northeast	2015	-0.41	0.0013
	2016	-0.40	0.0027
	2017	-0.41	0.0015
Northwest	2015	-0.26	0.0484
	2016	-0.33	0.0130
	2017	-0.23	0.0841
Centre	2015	-0.39	0.0027
	2016	-0.48	0.0002
	2017	-0.44	0.0006
South	2015	-0.58	<0.0001
	2016	-0.67	<0.0001
	2017	-0.7w	<0.0001
